# Heliox versus trimix in closed‐circuit rebreather diving: A comparative study of pulmonary and decompression stress in male divers

**DOI:** 10.14814/phy2.71129

**Published:** 2026-10-05

**Authors:** Emmanuel Gouin, Jérémy Orsat, Emmanuel Dugrenot, Rhiannon J. Brenner, Géraldine Leman, Eléonore Bettacchioli, Michael Theron, Marie‐Agnès Giroux‐Metges, Erwan L'Her, François Guerrero

**Affiliations:** ^1^ Univ Brest, Laboratory ORPHY EA 4324, IBSAM Brest France; ^2^ Anesthesiology, Perioperative and Intensive Care Units CHU La Cavale Blanche Brest France; ^3^ Divers Alert Network Durham North Carolina USA; ^4^ Lampe Joint Department of Biomedical Engineering The University of North Carolina and North Carolina State University Chapel Hill North Carolina USA; ^5^ Subaquatic Operational Research Team (ERRSO), Military Institute of Biomedical Research (IRBA) Toulon France; ^6^ Immunology and Immunotherapy Laboratory CHU Morvan Brest France; ^7^ Univ Brest, LBAI UMR 1227, Inserm Brest France; ^8^ Lung Function Unit CHU La Cavale Blanche Brest France; ^9^ Medical Intensive Care Unit CHU La Cavale Blanche Brest France; ^10^ Univ Brest, Laboratory LaTIM UMR 1101, Inserm Brest France

**Keywords:** decompression, inflammation, mixed‐gas, pulmonary function, technical diving, vascular bubbles

## Abstract

Closed‐circuit rebreather (CCR) and helium‐based breathing mixtures are widely used in technical diving. This study compared the effects of Heliox and Trimix on pulmonary function and decompression stress during CCR dives. Nineteen divers performed a standardized dive (50 ± 3 msw, 147 ± 10 min) using Heliox (16% O_2_/84% He; *n* = 9) or Trimix (16% O_2_/32% He/52% N_2_; *n* = 10). Pre‐ and post‐dive venous gas emboli (VGE), pulmonary function, lung ultrasound, heart rate variability, and inflammatory parameters were assessed. VGE were detected in all Heliox divers but in only 50% of Trimix divers. Heliox exhibited higher VGE counts 45 min post‐dive than Trimix (12 [IQR 3–32] vs. 0 [IQR 0–6], *p* = 0.03); this difference disappeared by 90 min. No clinically significant pulmonary function changes were observed. Lung diffusion capacity decreased moderately regardless of breathing gas (−6% ± 7%). Lung ultrasound revealed minor transient B‐line changes. Dive induced mild inflammatory responses, including neutrophilia and increased MRP8/14 in both groups. Heliox divers also showed transient complement activation alongside increased circulating platelets. CCR dives induced limited pulmonary stress, without gas‐dependent differences. Although Heliox may promote a higher post‐dive VGE burden and inflammatory responses, the relationship between these physiological effects and decompression sickness risk remains uncertain.

## 1 INTRODUCTION

Advances in specialized equipment and helium‐based mixed gases have driven rapid growth in recreational technical diving. Adding helium reduces nitrogen narcosis and gas density, enabling deeper dives (Gouin, Dugrenot, & Gardette, 2025; Mitchell & Doolette, 2013). The use of closed‐circuit rebreathers (CCR) offers substantial advantages in terms of gas consumption, optimized gas composition and decompression, and the delivery of warm, humidified breathing gas.

However, CCR induces a higher work of breathing (WOB) as compared to open‐circuit (OC) systems (Castagna et al., 2018). This increased respiratory effort associated with CCR use, combined with elevated gas density, may promote carbon dioxide (CO_2_) retention and potential pulmonary impairment (Anthony & Mitchell, 2015; Castagna et al., 2018; Dugrenot et al., 2021). Dive‐induced cardiopulmonary changes arise through multiple mechanisms, including hyperoxia, increased gas density, breathing circuit resistance, immersion‐induced blood shift, and decompression‐related alterations (Gouin et al., 2022; Tuominen et al., 2024).

Decompression stress remains a central concern in diving. Increased depth and bottom time result in greater inert gas uptake. During ascent, decreasing ambient pressure may lead to vascular gas emboli (VGE) formation, which are considered the primary mechanism underlying decompression sickness (DCS) (Mitchell, 2024). Beyond the classical bubble‐mechanical explanation, the complex pathophysiology of DCS involves, among other mechanisms, inflammatory processes and vascular impairment induced by diving, even in the absence of symptoms (Balestra et al., 2024; Mitchell, 2024; Vezzoli et al., 2024). While VGE are frequently present in asymptomatic divers, higher bubble grades are associated with an increased risk of DCS (Eftedal et al., 2007; Mitchell, 2024). The incidence of DCS appears higher in mixed‐gas diving, though clinical presentations are generally reported to be less severe than those observed in conventional recreational air diving (Gouin et al., 2026; Gouin, Monnot, et al. 2025; Tuominen et al., 2022). This suggests that the properties of breathing gases may influence decompression stress and the mechanisms underlying DCS.

Helium is lighter, highly diffusible, and has lower solubility than nitrogen. Some experimental studies, although conflicting, have reported that helium may have organ‐protective properties, including potential anti‐inflammatory effects and may reduce platelet activation (McIver et al., 1990; Oei et al., 2010). These properties could be beneficial for decompression and inert gas elimination.

Significant gaps remain in understanding the physiological stresses specific to CCR diving, particularly regarding cardio‐pulmonary function, decompression stress, and related biological responses. We hypothesized that a higher helium content, by reducing gas density and improving decompression kinetics, could mitigate diving‐induced physiological stress. This study aimed to compare the effects of two different helium‐based gas mixtures on pulmonary function and decompression stresses following prolonged CCR dives.

## 2 MATERIALS AND METHODS

This prospective interventional study was conducted in accordance with the Declaration of Helsinki and was approved by the Committee for Protection of Persons Est I, Dijon, France (2022‐A01639‐34). All participants provided a written informed consent.

### 2.1 Study population

Twenty males volunteered to participate. One diver was excluded due to substantial drysuit leakage and pronounced cold stress with respiratory symptoms, which could have markedly affected measurements unrelated to the breathing gas; resulting in an analysis on 19 divers. All were experienced mixed‐gas certified divers. The Table 1 summarizes the characteristics of the population. One Heliox diver was a smoker, and one Trimix diver had an arterial hypertension history. None had any respiratory conditions.

**TABLE 1:** Population characteristics.

	Overall	Heliox group (*n* = 9)	Trimix group (*n* = 10)	*p*‐value
Age (years) Mean ± SD	40 ± 11	39 ± 10	40 ± 12	0.8
BMI (kg.m^−2^) Mean ± SD	26.1 ± 3.7	26.5 ± 4.5	25.7 ± 3.1	0.6
Weekly physical activity (min) Median [Q1; Q3]	240 [120; 360]	240 [15; 420]	210 [120; 345]	0.9
Diving experience (years) Mean ± SD	22 ± 10	23 ± 11	20 ± 9	0.6
Tek diving experience (years) Mean ± SD	9 ± 5	10 ± 5	8 ± 5	0.2
Number of dives Mean ± SD	1844 ± 1071	2200 ± 1254	1523 ± 810	0.2
Number of trimix dive Median [Q1; Q3]	150 [50; 450]	150 [62; 550]	100 [41; 275]	0.3
DCS history *n* (%)	4 (21.1)	1 (11)	3 (30)	0.6

Abbreviations: BMI, Body Mass Index; DCS, decompression sickness.

Divers were instructed to refrain from any alcohol or coffee intake for 24 h before the dive and throughout the entire experimental period. None of the divers had dived in the previous 2 days.

### 2.2 Diving protocol

The protocol took place between September 2023 and September 2024 in the Bay of Brest, France. All dives were performed on the same site and with similar environmental conditions. Water temperature was 15.5°C ± 0.7°C. Divers used their own diving equipment, including personal dry suits and undergarments. They performed a similar recreational open water dive using back‐mounted counter lung units (rEvo Rebreather; *n* = 10, JJ‐CCR; *n* = 7, and APDiving Inspiration; *n* = 2). Ten divers breathed a Trimix blend (16% O_2_, 32% He, 52% N_2_), while the others opted for Heliox (16% O_2_, 84% He). The mean maximum depth was 50 ± 3 msw and the total time duration was 147 ± 10 min (Figure 1). Divers were requested to swim slowly and to avoid effort as much as possible. Decompression was conducted using the Bühlmann ZHL‐16C algorithm built into an integrated multi‐gas decompression computer, Petrel (Shearwater, Richmond, BC, Canada) or the 2020Vision computer (AP Diving Ltd., Helston, UK). Conservatism was set with a range of gradient factors (GF) to 30%–50% (low) and 70% (high). The oxygen partial pressure (PpO_2_) setpoint was set at 1.3 ATA during the bottom phase and could be increased to 1.4–1.5 ATA during decompression, in accordance with standard practice (Gouin, Monnot, et al. 2025). Oxygen exposure did not differ between groups, with CNS ‘clock’ values of 86% ± 17% for Heliox and 88% ± 11% for Trimix (*p* = 0.7), representing the proportion of the allowable oxygen toxicity exposure reached (Hoyt et al., 2025). Each buddy team included one Heliox and one Trimix diver following the same dive profile. Because decompression requirements were longer for Heliox due to differences in algorithm management, the ascent schedule was based on the Heliox obligations.

**FIGURE 1:**
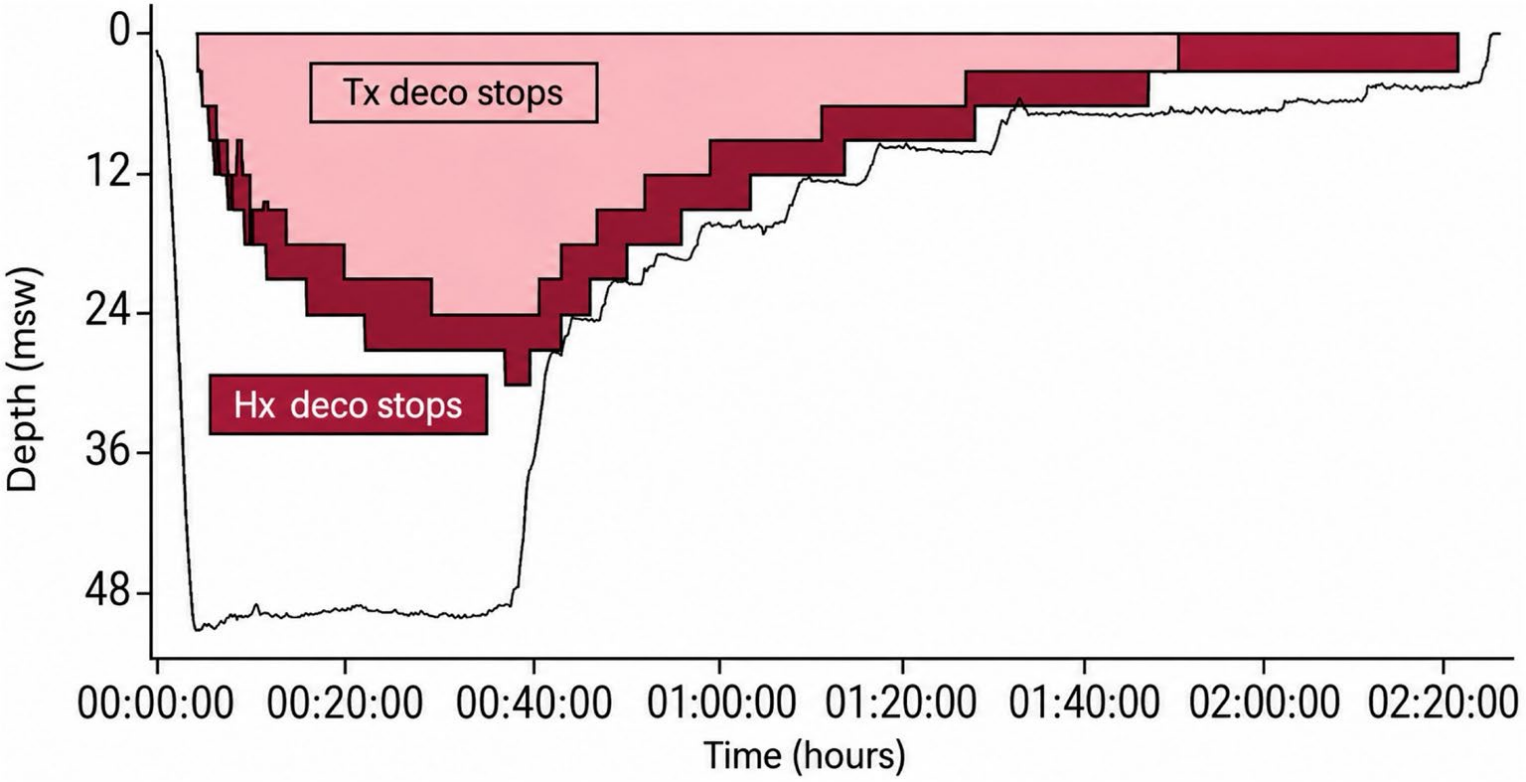
Example of a typical experimental dive profile with a maximum depth of 51 msw for 40 min bottom time. The black line represents the profile actually followed by the buddy team. The calculated decompression obligations according to the mixed gas were represented for Heliox (Hx) and Trimix (Tx) divers. The successive colored stepwise profile represents the decompression ceiling that must not be exceeded during ascent, with each step corresponding to a mandatory decompression stop. All diver performed their dive with the same planification parameters and similar dive profile that implies the Trimix diver stayed with his buddy after having completed his decompression obligation calculated by his connected computer.

### 2.3 Measurements

All measurements, unless otherwise stated, were performed close to the dive site, in a dedicated room, within 90 min prior to and after the dive, and they were repeated at 24 h. Divers wore comfortable clothing.

#### 2.3.1 Bubbles detection

VGE detection was used for decompression stress evaluation at 45 and 90 min after surfacing. A four‐chamber cardiac view centred on the right ventricle was obtained using 2D echocardiography (Sonosite M‐Turbo, Fujifilm Inc., Amsterdam, The Netherlands), with a 1–5 MHz phased array transducer, in the left lateral supine position without provocative manoeuvers. A quantitative assessment was performed using frame‐based bubble counting over 10 consecutive recorded cardiac cycles and was evaluated blindly by an experienced operator unaware of the gas mixture used (Germonpré et al., 2014).

#### 2.3.2 Functional and anatomical pulmonary monitoring

Pulmonary function test (PFT) was performed in a whole‐body plethysmography cabin (Vyntus BODY™; The Surgical Company B.V., Amersfoort, The Netherlands) at the University Hospital of Brest a few days before the dive (4 ± 3 days) and repeated within 150 min after surfacing. All measurements were obtained with the divers seated and at rest, under the supervision of specialized clinical staff. Both mobilizable and non‐mobilizable lung volumes were assessed, together with pulmonary flow rates and lung diffusion capacity for carbon monoxide (DLCO), which was systematically corrected for measured hemoglobin concentration. No provocative tests were performed. Results were expressed as absolute values and as percentages of predicted values, according to the Global Lung Initiative reference equations for Caucasian adults.

The anatomical pulmonary aeration was evaluated by a validated ultrasound quantitative approach using the same ultrasound device. Twelve thoracic areas were longitudinally scanned in supine position for a three‐second recording as previously described. The sum of B‐lines observed across all scanning sites was calculated afterwards by a trained operator (Castagna et al., 2018; Gouin et al., 2022). Additionally, pulse oximetry saturation (SpO_2_) was recorded by a dedicated oximetry module (MIR Medical International Research Srl, Rome, Italy).

The respiratory symptoms were assessed through a targeted questionnaire including shortness of breath, thoracic pain, cough, feeling of sputum, and thoracic pain.

#### 2.3.3 Hemodynamic parameters and hydric balance

Non‐invasive arterial blood pressure (ABP) and heart rate (HR) were assessed by Omron M7 IntelliSenseTM monitor (Omron Healthcare Co., Kyoto, Japan) after 10 min of rest in the seating position. For safety reasons, divers were not restricted in their fluid intake during the protocol. After emptying their bladder, subjects were weighed on an electronic device (Tanita®, Hoofddorp, The Netherlands; precision ±20 g) pre‐ and post‐dive, and all fluid intakes were quantified between measurements. The hydric balance (HB) was estimated as HB = [Weight post – Weight pre] – Fluid intake.

#### 2.3.4 Cardiac autonomic function

A chest belt sensor Polar H10 (Polar Electro Oy, Kempele, Finland) was used to record the short‐term R‐to‐R intervals at a 1000 Hz sampling frequency. All data were transferred to a dedicated computer and analyzed using Kubios HRV Scientific Lite 4.1.0 (Kubios Oy, Kuopio, Finland). The automatic artifact correction function of the program was used to correct data corruption for each subject before analysis. Heart Rate Variability (HRV), including time‐domain, frequency‐domain, and non‐linear parameters, were subsequently computed. In addition, two composite indexes reflecting parasympathetic nervous system (PNS) and sympathetic nervous system (SNS) activity were calculated (Lundell et al., 2021; Tarvainen et al., 2014).

#### 2.3.5 Laboratory analysis

Venous blood samples were taken from an antecubital arm vein into vacuum tubes containing EDTA (Greiner Bio‐One, Monroe, NC, USA). Hemogram assays were measured pre‐dive and post‐dive in whole blood using the XN‐9100‐2‐A™ automated analyzer (Sysmex Europe SE, Norderstedt, Germany).

Plasma was obtained by centrifuging at 1500 G (2500 rpm) for 15 min at 20°C, within 1 h after withdrawal. The plasma samples were stored frozen at −80°C in multiple aliquots prior to thawing. Complement analysis was performed with specific CH50, C3c, and C4 kits (NK095.OPT; NK023.OPT; NK025.OPT) using an Optilite automated analyzer (Binding Site, ThermoFisher Scientific, Saint‐Égrève, France). Inflammatory biomarkers were measured using a high‐sensitivity LEGENDplex™ Human Vascular Inflammation Panel 1 Standard Mix with antibody‐coated beads (Cat. No. 740554; BioLegend, Inc., San Diego, CA, USA), comprising C‐reactive protein (CRP; Cat. No. 740558), neutrophil gelatinase‐associated lipocalin (NGAL; Cat. No. 740557), vascular cell adhesion molecule‐1 (VCAM‐1; Cat. No. 740564), myeloid‐related protein 8/14 (MRP8/14; Cat. No. 740556), intercellular adhesion molecule‐1 (ICAM‐1; Cat. No. 740563), cystatin C (Cat. No. 740566), and myeloperoxidase (MPO; Cat. No. 740561). A LEGENDplex™ Custom Human Panel 2300 (No. 900007734; BioLegend, Inc., San Diego, CA, USA), containing custom Human 5‐plex premixed beads (No. P1782), was used to quantify interleukin‐6 (IL‐6), interleukin‐8 (IL‐8), interleukin‐1β (IL‐1β), C‐C motif chemokine ligand 2 (CCL2), and epidermal growth factor (EGF).

All assays were performed in duplicate in accordance with the manufacturer's instructions. Duplicates showing a coefficient of variation greater than 30% were interpreted with caution, as recommended by the manufacturer. When such values also exceeded three times the median absolute deviation of distribution, they were considered unreliable outliers and were excluded from the analysis (Kannan et al., 2015).

### 2.4 Statistical analysis

Data are presented as mean ± SD for normally distributed variables, or as median IQR [Q1; Q3]. The normality of data distribution was assessed by the Shapiro–Wilk test. Categorical unpaired variables were compared using Fisher's exact test. Within‐group comparisons (Helium vs. Trimix) were performed using the unpaired *t*‐test for normally distributed continuous variables, or the Mann–Whitney test otherwise. Intra‐individual comparisons across time points for bubble measurements were conducted using the paired Wilcoxon test. For analyses involving repeated time points and group effects, a two‐way repeated‐measures ANOVA followed by Tukey's post hoc multiple‐comparison test was applied when normality assumptions were met for all groups. If normality was violated in at least one group, analyses were conducted using the nonparametric Friedman test followed by Dunn's post hoc test. Statistical analysis was conducted using GraphPad Prism v10.4.0 (GraphPad Software Inc., San Diego, CA, USA). A *p*‐value <0.05 was considered statistically significant.

## 3 RESULTS

### 3.1 Bubbles formation

None of the divers developed symptoms of DCS. VGE were detected in all Heliox divers, whereas they were observed in five (50%) Trimix divers. Figure 2 depicts the bubbles analyses. At 45 min post‐dive, Heliox divers exhibited higher VGE counts than Trimix divers, with respectively 12 [3–32] versus 0 [0–6] bubbles per 10 cardiac cycles (*p* = 0.03). This difference was no longer significant at 90 min (*p* = 0.3). The maximum bubble count measured did not differ between divers with and without a history of DCS, with 11 [9–18] versus 5 [0–25] bubbles respectively; *p* = 0.8.

**FIGURE 2:**
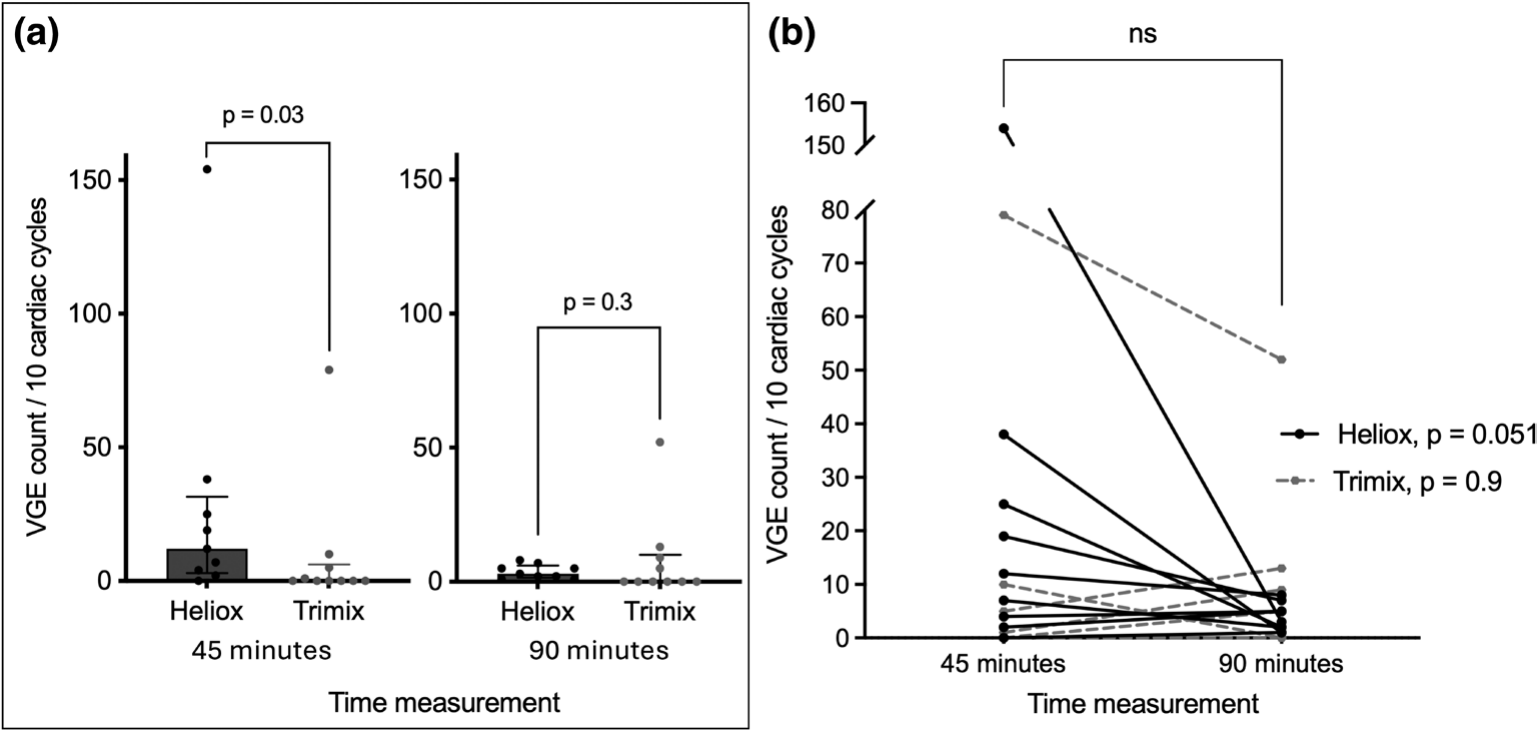
Frame‐based vascular gas emboli (VGE) count at 45‐min and 90‐min post‐dive. Between‐group comparisons (a) at each time point were performed using the Mann–Whitney test. Results are presented as median [IQR]. Intra‐individual comparison (b) between 45 and 90 min using the paired Wilcoxon matched‐paired test for each group. VGE, Vascular gas emboli.

### 3.2 Respiratory assessment

One Trimix diver experienced a transient post‐dive cough that resolved rapidly, while no other respiratory complaints were reported. No significant changes in PFT parameters were observed following the dive, with the notable exception of FEF50%. Post‐hoc analysis revealed a significant decrease specifically within the Heliox group. Regarding gas exchange, a significant time effect on DLCO was found (*p* = 0.001), independently of gas type or gas*time interaction. Further post‐hoc analysis specified that this decline was statistically significant only for the Trimix group, which showed a mean post‐dive reduction of −7% ± 5% (compared to −5% ± 9% for Heliox). Although a significant time effect was observed for SpO_2_ (*p* = 0.02), post hoc analyses did not reveal any significant intra‐group differences (Table 2).

**TABLE 2:** Pulmonary functional test parameters pre‐dive and post‐dive.

Variable	*p*‐values			Heliox (*n* = 9)		Trimix (*n* = 10)	
	Gas	Time	Gas*time	Pre	Post	Pre	Post
VC max (L)	0.6	0.3	0.4	5.7 ± 0.9	5.7 ± 0.8	6 ± 1	6 ± 1
% Theo	0.5	0.3	0.6	107 ± 13	107 ± 13	110 ± 6	111 ± 5
FCV (L)	0.4	0.9	0.08	5.6 ± 0.9	5.5 ± 0.8	5.8 ± 1	5.9 ± 1
% Theo	0.3	0.8	0.047	105 ± 12	103 ± 14	108 ± 6	110 ± 5
FEV1 (L/s)	0.5	0.7	0.1	4.3 ± 0.8	4.2 ± 0.6	4.5 ± 0.9	4.5 ± 0.9
% Theo	0.4	0.6	0.1	99 ± 14	98 ± 13	103 ± 10	104 ± 10
FEV1/CVF (%)	0.8	0.9	0.8	77 ± 4	77 ± 6	76 ± 4	76 ± 4
% Theo	0.9	0.9	0.6	95 ± 6	95 ± 9	95 ± 6	94 ± 5
PEF (L/s)	0.2	0.6	0.03	10 ± 1.2	9.8 ± 1.4	10.6 ± 1.6	10.9 ± 1.4*
% Theo	0.2	0.6	0.03	105 ± 14	103 ± 16	112 ± 19	115 ± 15
FEF75% (L/s)	0.5	0.3	0.3	7.8 ± 2	7.5 ± 1.8	8.3 ± 2	8.3 ± 2
% Theo	0.4	0.3	0.3	94 ± 24	90 ± 22	100 ± 22	100 ± 22
FEF50% (L/s)	0.6	0.04	0.4	4.5 ± 1.2	4.2 ± 0.9*	4.7 ± 1.4	4.6 ± 1.4
% Theo	0.5	0.05	0.5	109 ± 30	102 ± 29	116 ± 31	112 ± 29
FEF25% (L/s)	0.9	0.96	0.6	1.6 ± 0.7	1.6 ± 0.6	1.6 ± 0.6	1.6 ± 0.7
% Theo	0.8	0.9	0.4	100 ± 35	105 ± 33	101 ± 30	97 ± 34
RV (L)	0.02	0.5	0.9	1.3 ± 0.4	1.3 ± 0.4	1.3 ± 0.4	1.3 ± 0.4
% Theo	0.01	0.5	0.97	85 ± 17	82 ± 20	65 ± 21	62 ± 17
TLC (L)	0.8	0.8	0.7	7.4 ± 0.9	7.4 ± 0.9	7.3 ± 1	7.3 ± 1.2
% Theo	0.7	0.7	0.9	101 ± 9	100 ± 10	99 ± 7	98 ± 8
DCLO mmol/(min*kPa)	0.4	0.001	0.4	9.53 ± 1.09	9.1 ± 1.5	10.18 ± 1.3	9.47 ± 1.39**
% Theo	0.3	0.002	0.4	89 ± 10	84 ± 12	95 ± 10	89 ± 10**
KCO mmol/(min*kPa*L)	0.7	0.007	0.7	1.4 ± 0.17	1.33 ± 0.18	1.44 ± 0.13	1.35 ± 0.1*
% Theo	0.7	0.004	0.6	90 ± 12	85 ± 11	92 ± 9	86 ± 8*
SpO_2_ (%)	0.3	0.02	0.8	98 ± 0.8	97.1 ± 1.6	97.5 ± 0.8	96.8 ± 0.9

*Note*: Results are mean ± SD. Two‐way repeated measures ANOVA followed by Tukey post‐hoc multiple comparisons test is expressed versus predive with *p < 0.05 and **p < 0.01.

Abbreviations: %Theo, percentages of predicted values; DLCO, lung diffusion capacity for carbon monoxide; FCV, forced vital capacity; FEF, forced expiratory flow; FEV1, forced expiratory volume exhaled in the first second; KCO, carbon monoxide transfer coefficient; PEF, peak expiratory flow; RV, residual volume; SpO_2_, pulse oximetry saturation; TLC, total lung capacity; VC max, maximal vital capacity.

Lung ultrasound depicted a temporal variation in B‐lines counts within the Heliox group (*p* = 0.036). However, despite this global trend, post‐hoc analysis did not identify any significant intra‐group differences at specific time points (Figure 3).

**FIGURE 3:**
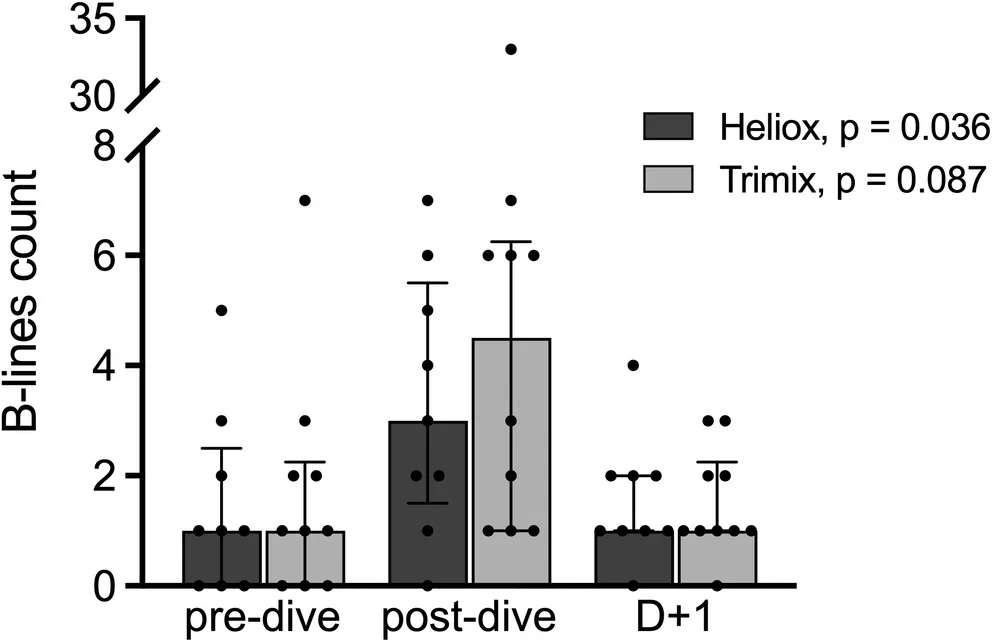
Pulmonary ultrasound B‐lines count. Results are presented as median [IQR]. Dunn's post‐hoc multiple‐comparison test was non‐significant, showing no significant post‐dive increase in the number of B‐lines.

### 3.3 Hemodynamic and cardiac autonomic function

Hemodynamic data are summarized in Figure 4. A significant time effect on ABP was observed, with values decreasing after the dive and remaining significantly below baseline at D + 1. Hydric balance was −1.8 ± 0.8 L in Heliox divers and −2.0 ± 1.0 L in Trimix divers, with no significant difference between groups (Figure 5). The weight returned to its baseline value at D + 1. Hematocrit levels were unchanged after the dive in both groups with 44.2% ± 2.6% pre‐dive versus 44% ± 2.1% post‐dive in the Heliox group and 44.2% ± 3.4% versus 45% ± 3.7% in the Trimix group, respectively. No effect of time (*p* = 0.7), gas mixture (*p* = 0.5), or gas*time interaction (*p* = 0.3) was observed.

**FIGURE 4:**
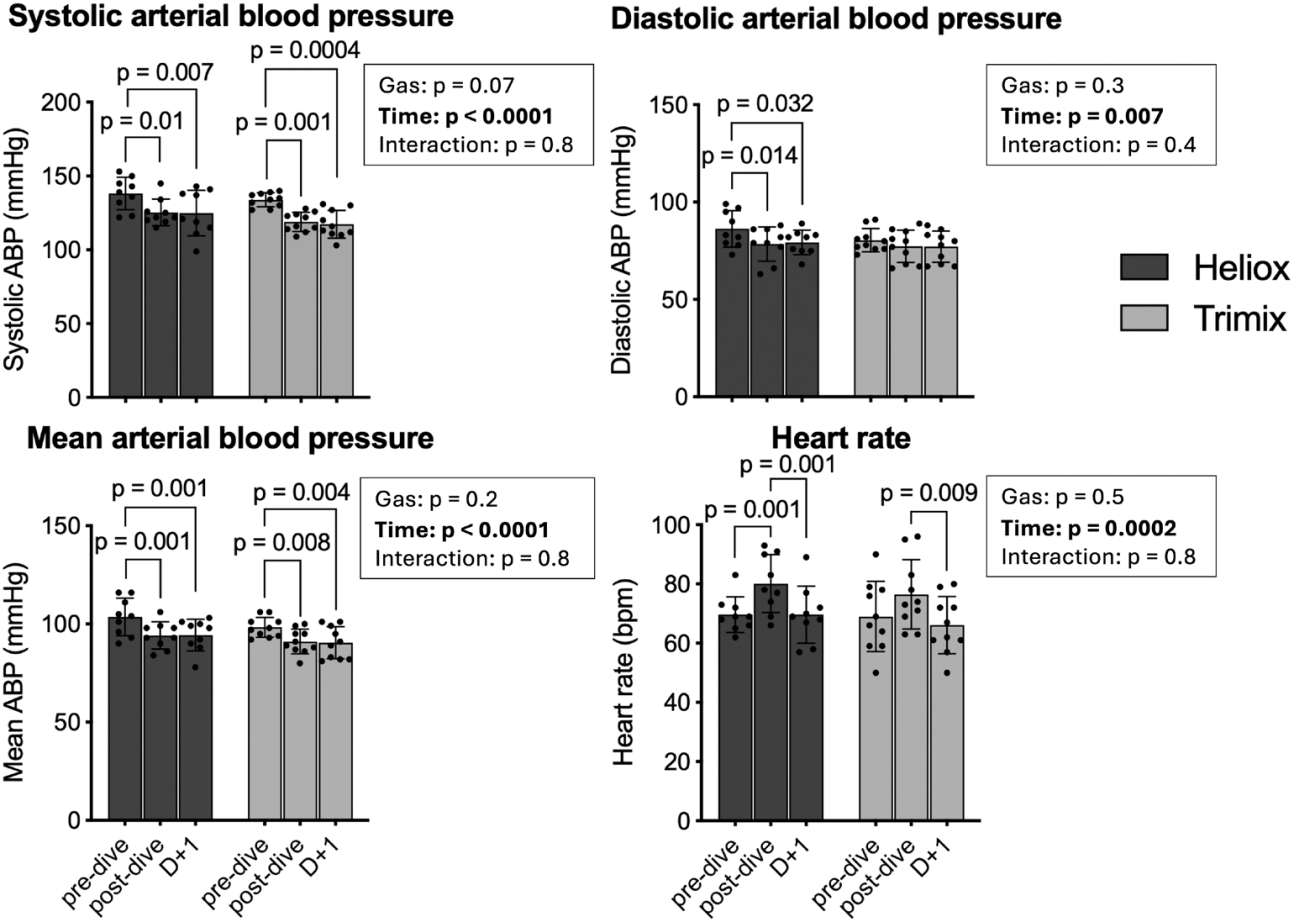
Hemodynamic parameters. Results are presented as mean ± SD. Two‐way repeated measures ANOVA followed by Tukey post‐hoc multiple comparisons test represented versus predive.

**FIGURE 5:**
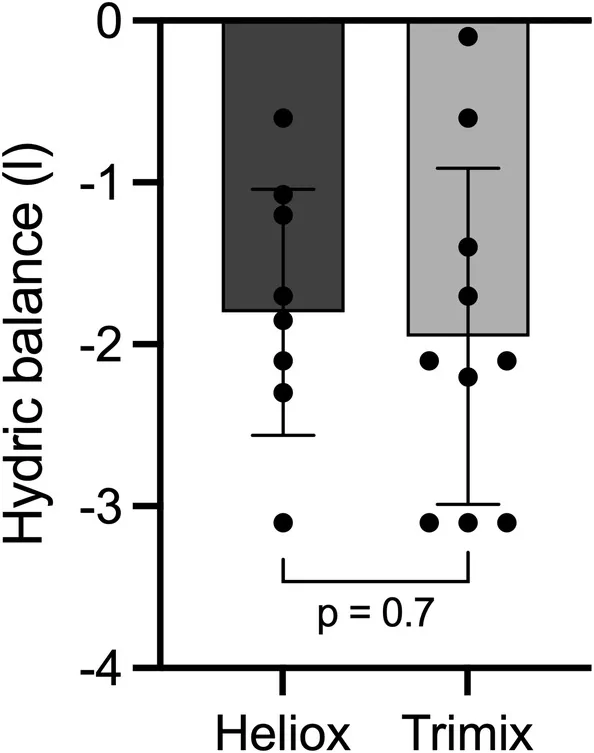
Hydric balance calculation between pre‐dive and post‐dive. Results are presented as mean ± SD. Group comparison used unpaired *t*‐test.

Mean RR decreased after the dive and returned to baseline at D + 1 in the Heliox group (*p* = 0.03), whereas in the Trimix group the change was observed only between post‐dive and D + 1 (*p* = 0.04). However, none of the other time‐ or frequency‐domain parameters showed significant changes over time. All HRV data are provided in Table S1 (available at: https://doi.org/10.6084/m9.figshare.32622141). Sample Entropy (SampEn) decreased after the dive and differed significantly between pre‐dive measurements in the Heliox group, whereas the other entropy indices showed significant differences only between post‐dive and D + 1 measurements. Figure 6 depicts Entropy and ANS composite indices.

**FIGURE 6:**
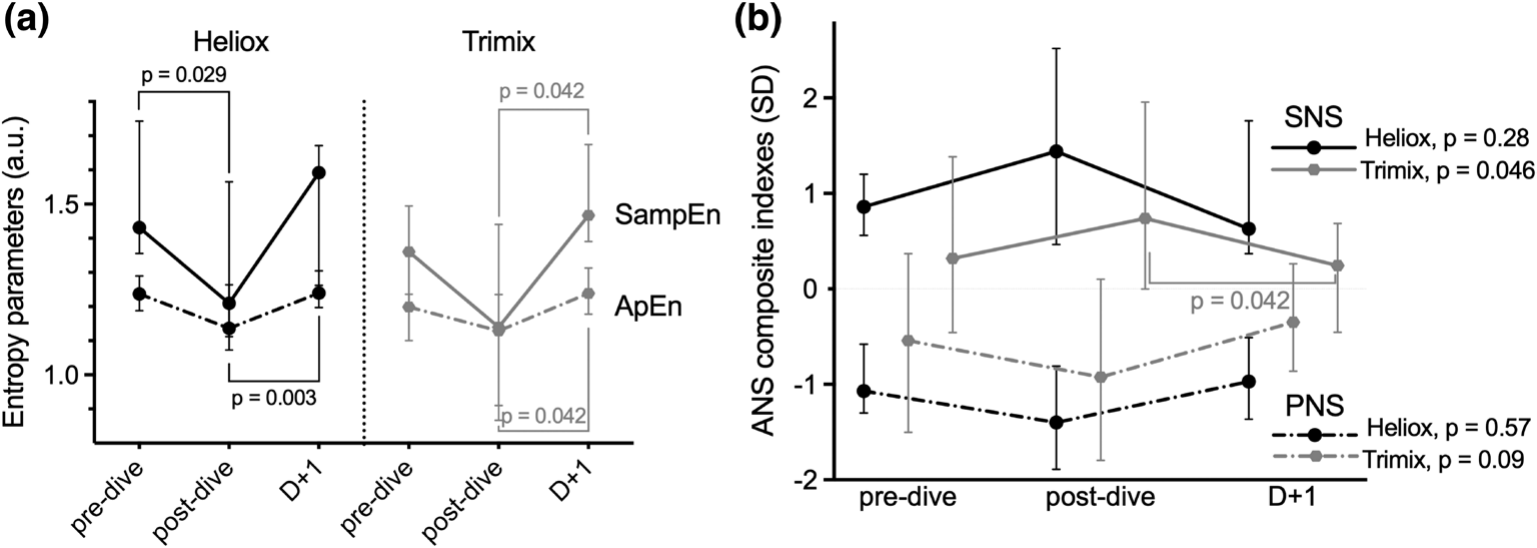
Entropy parameters (a) and autonomic nervous system (ANS) composite indexes (b) measured by HRV. Results are presented as median [IQR]. Intra‐group comparisons were assessed using the Friedman test followed by Dunn's post‐hoc test. ApEn, approximate entropy; SampEn, sample entropy; SNS, sympathetic nervous system; PNS, Parasympathetic nervous system composite indexes.

### 3.4 Biological inflammatory response

The hemogram showed a significant time effect, with no significant gas effect or gas*time interaction on any parameter (Table 3). The increase in platelet count reached statistical significance in post‐hoc analyses only within the Heliox group. A neutrophil‐driven increase in leukocyte counts was observed in both groups after the dive. Analysis of the complement system revealed a significant time effect and time*gas interaction for total hemolytic complement activity (CH50) and complement component C3c. Post‐hoc analysis showed a significant decrease in CH50 following the dive exclusively in the Heliox group, with levels returning to baseline by D + 1, accompanied by a concurrent reduction in C3c (Table 3).

**TABLE 3:** Temporal evolution of the hemogram and the complement system.

Variable	*p*‐value			Heliox			Trimix		
	Gas	Time	Gas*time	Pre	Post	D + 1	Pre	Post	D + 1
RBC (T.l^−1^)	0.98	0.03	0.3	5 ± 0.3	5.1 ± 0.3	—	5 ± 0.4	5.1 ± 0.4*	—
PLT (G.l^−1^)	0.4	0.005	0.6	225 ± 39	252 ± 43*	—	243 ± 32	262 ± 40	—
Leuco (G.l^−1^)	0.4	<0.0001	0.97	6.9 ± 1.3	8.8 ± 2**	—	6.3 ± 1.2	8.2 ± 2.2**	—
Neutro (/mm^3^)	0.4	0.0002	0.9	3803 ± 1202	6039 ± 1946**	—	3318 ± 1158	5414 ± 2067**	—
CH50 (U.l^−1^)	0.9	0.01	0.1	60 ± 7.6	56.7 ± 6.9*^,^^†^	59.4 ± 8.1	59.1 ± 9.6	57.7 ± 8.4	57.4 ± 6.9
C3c (g.l^−1^)	0.1	0.003	0.001	1.14 ± 0.14	1.1 ± 0.13**	1.12 ± 0.15	1.04 ± 0.15	1.04 ± 0.14^†††^	0.99 ± 0.12**
C4 (g.l^−1^)	0.6	0.2	0.053	0.22 ± 0.05	0.22 ± 0.05	0.22 ± 0.05	0.24 ± 0.09	0.24 ± 0.09	0.23 ± 0.09

*Note*: Results are mean ± SD. Two‐way repeated measures ANOVA followed by Tukey post‐hoc multiple comparisons test represented versus predive with *p < 0.05 and **p < 0.01. Differences versus D + 1 are expressed with ^†^ *p* < 0.05 and ^†††^ *p* < 0.001.

Abbreviations: C3c, complement component 3c; C4, complement component 4; CH50, total hemolytic complement activity; Leuco, total leucocytes count; Neutro, neutrophil count; PLT, platelet count; RBC, red blood count.

There was a significant increase in MRP8/14 after the dive in both groups, and a slight increase in IL‐6 was observed only in the Trimix group (Figure 7). CRP levels increased with a delayed response in the Heliox group. Post hoc analysis revealed a significant difference between post‐dive and D + 1 values in this group (*p* = 0.04), whereas no statistically significant change was observed in the Trimix group. Conversely, CCL2 levels showed a non‐significant increase immediately post‐dive, followed by a significant decrease at D + 1 compared with baseline values in the Heliox group (*p* = 0.008). Likewise, no significant changes were detected for IL‐1β, IL‐8, MPO, EGF, NGAL, ICAM‐1, or VCAM‐1 in either group (Table S2; available at: https://doi.org/10.6084/m9.figshare.32622141). No significant correlation was found between the maximal VGE count and the evolution pre‐ and post‐dive of the biomarkers using the Spearman correlation test (detailed results not presented; *p* > 0.05).

**FIGURE 7:**
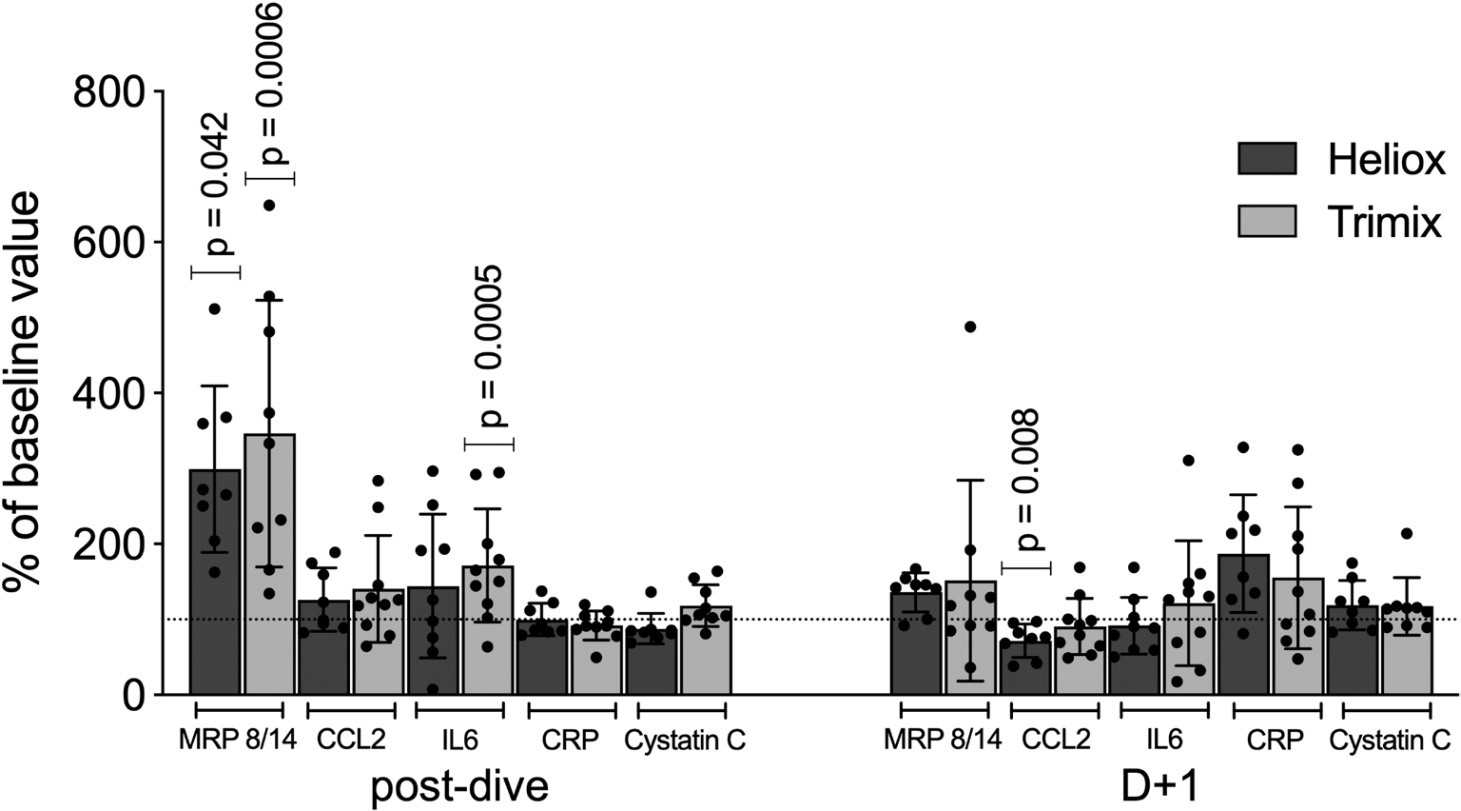
Percentage change from baseline in inflammatory biomarkers showing significant variation during the follow‐up period assessed by Friedman test (refer to Tables S2 for complete data). Results are expressed as percentage of baseline value (mean ± SD). Only statistically significant changes versus baseline are shown, as determined by Dunn's post‐hoc comparisons of absolute post‐dive and D + 1 measurements.

## 4 DISCUSSION

The acute physiological responses were limited in magnitude and appeared primarily driven by the dive itself rather than by breathing gas composition following a prolonged helium‐based CCR dive. Because both groups were exposed to comparable diving profiles, environmental conditions, equipment, and oxygen exposure, any slight differences between groups are likely attributable to the composition of the inert gases (helium/nitrogen versus helium alone), induced decompression stress, and subsequent mechanisms.

Decompression strategies in bounce mixed‐gas diving remain unvalidated and subject of debate (Doolette & Mitchell, 2013; Gouin, Monnot, et al., 2025). In this study, dive profiles followed user‐selected GF based on manufacturer presets and common practices (Gouin, Monnot, et al., 2025). Heliox divers demonstrated a significantly higher bubble count at early time points (45 min), which declined rapidly at 90 min. All Heliox divers exhibited VGE, whereas only 50% of Trimix divers did, which may have influenced the observed responses, particularly at the biological level. The physicochemical properties of inert gas influence bubble dynamics, with helium promoting both accelerated bubble formation and faster washout due to its relatively low solubility coefficient (Balestra et al., 2022; Ljubkovic, Marinovic, et al., 2010; Tuominen et al., 2024). Previous research indicates that bubble peaks typically occur about 45 min after helium‐rich mixtures dives (Balestra et al., 2022), whereas peaks may appear later with nitrogen‐based mixtures. However, the limited number of measurement points, imposed by logistical constraints, may have decreased the temporal resolution and thereby limited detection of the true peak in bubble formation (Møllerløkken et al., 2016). Comparing the effect of breathing mixtures is challenging while each mixture provides a different decompression dose, for the same dive parameters. The lower VGE levels observed in the Trimix group may reflect a longer in‐water decompression duration than that prescribed by dive computers, thereby reducing bubble formation. However, because the algorithms embedded in commercial dive computers induce a non‐physiologically relevant decompression penalty for helium use, and given that Navy Experimental Diving Unit (NEDU) comparisons of similar gas mixes have demonstrated a potentially increased risk associated with Trimix (Doolette et al., 2015), it can be inferred that both groups followed highly conservative decompression profiles. Ultimately, few divers in any group exhibited high VGE grade ≥3. A more aggressive protocol with reduced in‐water decompression time might have produced greater variations in the measured parameters and could have led to more harmful effects. Post‐dive fluid loss was substantial (−1.9 ± 0.9 L), but no relation was observed with VGE. This aligns with previous studies reporting immersion‐related fluid loss during diving (Castagna et al., 2015; Dugrenot et al., 2021; Tuominen et al., 2025). Despite this weight loss, hematocrit remained unchanged, indicating that compensatory fluid shifts from the extravascular to the vascular compartment were sufficient to maintain intravascular volume. Post‐dive dehydration has been suggested to increase DCS risk (Fahlman & Dromsky, 2006), and prehydration may favorably influence VGE production (Gempp et al., 2009).

We initially hypothesized that higher breathing gas density might increase WOB and thereby exacerbate pulmonary alterations. At maximum depth, gas density reached 5.8 g/L in the Trimix group and 2.7 g/L in the Heliox group, both within recommended limits (Anthony & Mitchell, 2015). Overall, regardless of breathing gas, the observed pulmonary stress was minimal and without clinical significance. Nevertheless, many technical divers operate beyond these gas density thresholds, potentially exacerbating respiratory loading (Gouin, Monnot, et al., 2025). Post‐dive B‐lines are common, transient markers of extravascular lung water and have been reported after both deep OC dives (Ljubkovic, Gaustad, et al., 2010; Marinovic et al., 2010), and shallow CCR dives (Castagna et al., 2018; Gouin et al., 2022; Martinez‐Villar et al., 2022). Multiple mechanisms may contribute to these asymptomatic cardiopulmonary changes (Castagna et al., 2017). Exercise intensity and VGE may increase pulmonary arterial pressure (Castagna et al., 2018; Marabotti et al., 2013; Marinovic et al., 2010). Additionally, CCR diving has been linked to altered left ventricular diastolic and atrial function (Martinez‐Villar et al., 2022; Tuominen et al., 2024), potentially promoting pulmonary congestion and stress on the alveolo‐capillary membrane. Substantial interindividual variability in B‐line formation complicates attributing effects to breathing gas composition (Gouin et al., 2022; Peacher et al., 2010). Susceptibility likely depends on individual pulmonary compliance and right ventricular responses to immersion (Castagna et al., 2023; Druelle et al., 2024).

Previous studies reported no spirometric impairment after single open‐circuit dives up to 65 m, although environmental conditions such as depth and water temperature may influence outcomes (Dujic et al., 2005; Ljubkovic, Marinovic, et al., 2010; Tetzlaff et al., 2001). Despite the additional respiratory load and oxygen exposure associated with CCR diving, no significant post‐dive spirometric changes have been observed after CCR dives to 15–70 m at typical technical diving PpO_2_ levels (1.2–1.6 ATA) (Bosco et al., 2018; Fock et al., 2013; Gouin et al., 2022). Consistent with these findings, our study showed no significant changes in lung volumes or flow rates, except for a slight reduction in FEF50% in Heliox divers, possibly related to cold‐induced small airway reactivity due to helium's high thermal conductivity. In contrast, following repetitive deeper CCR dives (90–120 msw), a gradual FVC decrease was observed (Dugrenot et al., 2021). Exposure to greater depths, as well as the subsequent decompression phase, may have contributed to this alteration. In addition, other potential confounding factors, such as wetsuit use and increased physical exertion at depth, may have further reduced pulmonary compliance and increased inspiratory effort; factors absent in the present study (Castagna et al., 2018; Stevens & Smart, 2025). A moderate decrease in DLCO is commonly observed after diving and has been linked to oxygen exposure and bubble formation (Thorsen et al., 1993; van Ooij et al., 2011). In our study, the DLCO reduction (−6% ± 7%) remained within physiological limits and was not influenced by either breathing gas composition or VGE burden.

Conventional HRV indices remained unchanged in our study. The cardiac autonomous nervous system is challenged by various factors, reflecting hemodynamic constraints and responding to decompression stress (Schirato et al., 2020). Diving is typically associated with increased parasympathetic modulation during immersion, followed by sympathetic predominance during recovery, although post‐dive HRV findings remain inconsistent (Chouchou et al., 2009; Dugrenot et al., 2021; Lundell et al., 2021; Noh et al., 2018; Schirato et al., 2018, 2020). Diver experience may also enhance autonomic regulation (Chen et al., 2025). In our study, the high level of expertise of our participants may have contributed to the relatively stable HRV parameters observed. In addition, as previously suggested, decompression stress associated with the present dive profile appears to have been moderate. A modest entropy variation was observed, reflecting the heart rate complexity and adaptability, but its isolated interpretation is uncertain.

Inflammatory activation and bubble formation are closely interconnected processes in decompression stress and in the pathophysiology of DCS (Mitchell, 2024; Vezzoli et al., 2024). VGE have been shown to trigger thrombo‐inflammatory responses including complement activation, leukocytosis, and cytokine release (Bigley et al., 2008; Orsat et al., 2026; Pontier et al., 2012; Storm et al., 2022). Consequently, acute changes in inflammatory markers have been proposed as indicators of decompression quality (Spisni et al., 2017). CCR diving may further influence these processes by increasing oxygen exposure and the use of mixed gases (Arya et al., 2023; Balestra et al., 2024; Bosco et al., 2018; Vezzoli et al., 2024).

In our study, a modest increase in inflammatory biomarkers, along with decreased complement levels suggestive of its activation, was observed. Complement C3 undergoes spontaneous hydrolysis that can be amplified by external stimuli such as bubbles, activating the alternative pathway and leading to decreased C3c levels (Storm et al., 2021, 2022). While no significant relationship was found between C3c and bubbling intensity, C3c and CH50 significantly decreased pre‐ to post‐dive in Heliox divers, who also initially exhibited significantly higher bubble burden than Trimix divers. Interestingly, platelet counts increased significantly in the Heliox group. This finding contrasts with most air‐diving studies, which have reported reductions in platelet counts, generally attributed to bubble‐induced platelet activation and consumption (Lambrechts et al., 2013; Pontier et al., 2012). However, some studies found no change (Cialoni et al., 2022; Perovic et al., 2017), including in trimix diving (Olszanski et al., 2010). Importantly, this increase does not preclude platelet activation; rather, it may indicate that acute stress‐induced platelet mobilization and demargination exceeded consumption. In addition, although circulating VGE are known to activate both platelets and the complement system at the gas–liquid interface, experimental studies have suggested that helium bubbles induce less irreversible platelet aggregation and subsequent thrombocytopenia than nitrogen bubbles (Olszanski et al., 2010). Platelets also play a key role in inflammatory regulation, and thrombocytosis is frequently observed in inflammatory processes, which may be triggered in this context (Ludwig et al., 2022). Post‐dive leukocytosis was primarily driven by neutrophilia in both groups. Neutrophil demargination and activation after diving has been consistently reported and may interact with activated platelets (Cialoni et al., 2022; Dumić et al., 2022; Perovic et al., 2017; Thom et al., 2013). In our study, pro‐inflammatory cytokines IL‐6, IL‐8, and IL‐1β did not increase significantly after the dive, except for a slight elevation of IL‐6 in the Trimix group. This is consistent with previous technical diving studies (Rocco et al., 2021; Spisni et al., 2017). A recent experimental study investigating mild pressure exposure to nitrogen or helium mixtures reported a comparable dynamic affecting IL‐6 (Balestra et al., 2024). These cytokines require transcriptional responses that typically occur over 4–6 h, which may explain the absence of an early increase; moreover, their short half‐life may preclude detection at D + 1. In contrast, myeloid factors stored in neutrophil granules are rapidly released. The neutrophil‐derived mediator MRP8/14 increased in both groups, likely reflecting neutrophil activation occurring to a similar extent in each group (Pruenster et al., 2015). CCL2 is released during inflammation to recruit myeloid cells, particularly in response to vascular injury (Carr et al., 1994). The variation observed only in the Heliox group likely reflects greater mechanical stress, which could hypothetically be associated with higher VGE load. A similar trend has been reported after Trimix diving, depending on the decompression model (Spisni et al., 2017). However, VCAM‐1 levels did not change significantly, suggesting limited endothelial activation and potentially subtle differences in compensatory vascular responses.

Paradoxically, the oxy‐inflammatory and vascular response may be more pronounced with higher helium content, possibly related to a greater bubble burden, without being counterbalanced by the anti‐inflammatory properties traditionally attributed to helium. While helium preconditioning has been found to reduce vascular injury in animal models (de Deken et al., 2016), as well as reduce inflammatory response and leukocyte adhesion among damaged tissues under oxidative stress (Lucchinetti et al., 2009; Smit et al., 2013), it has also been found to increase the release of membrane microparticles, potentially indicating increased cellular stress after prolonged exposure in humans (Balestra et al., 2024; Smit et al., 2015). Overall, our findings are in line with recent observations by Balestra, who reported that exposure to nitrogen or helium at low pressures (1.45 ATA) elicited distinct inflammatory responses, with helium potentially promoting a more pro‐inflammatory profile (Balestra et al., 2024).

### 4.1 Limitations

This study has several limitations. Although conducted under real‐world technical diving conditions, the sample size was relatively small. The lack of randomization for gas selection and the non‐crossover design, with each diver performing only one condition, may have allowed unmeasured confounding factors to influence the results. In particular, the groups may have differed in their inherent susceptibility to VGE formation due to the substantial inter‐ and intra‐individual variability in bubble production. In addition, no control group without helium (i.e. air diluent) was tested. The gas mixtures were designed to be as different as possible while remaining consistent with current recommendations regarding gas density and narcosis potential at the target depth, which precluded the use of air at this depth.

We attempted to minimize bias related to environmental conditions (e.g., seawater temperature, current, and use of a similar dive site) and procedural parameters (e.g., low physical exertion, controlled oxygen exposure, and standardized dive profile). Matching immersion durations between groups inevitably resulted in decompression times that substantially exceeded those calculated by the dive computer, which may have influenced physiological responses. This may have affected the magnitude of decompression stress, particularly in the Trimix group, whose divers underwent longer decompression than prescribed, potentially contributing to the low VGE burden observed. VGE measurements were performed at only two time points; although these were selected based on the expected timing of the VGE peak, this limited sampling may have affected the results and precludes characterization of the full temporal dynamics of VGE. Finally, the dive profile may not have been sufficiently strenuous to elicit marked physiological changes in both groups.

## 5 CONCLUSION

Subclinical pulmonary and decompression stresses were not significantly influenced by the type of helium‐based breathing mixtures. However, a high helium content may lead to a higher transient post‐dive VGE burden, which (contrary to observations reported outside the hyperbaric setting) could hypothetically contribute to a more pronounced inflammatory response. The relationship between the physiological effects of helium and both the risk and clinical presentation of DCS remains unclear, and further research is warranted to better elucidate this association.

## AUTHOR CONTRIBUTIONS

**Emmanuel Gouin:** Conceptualization; data curation; formal analysis; investigation; methodology; project administration; validation; visualization. **Jérémy Orsat:** Investigation; methodology; validation. **Emmanuel Dugrenot:** Conceptualization; investigation; methodology; validation. **Rhiannon J. Brenner:** Investigation; validation. **Géraldine Leman:** Formal analysis; investigation; validation. **Eléonore Bettacchioli:** Investigation; validation. **Michael Theron:** Formal analysis; investigation; validation. **Marie‐Agnès Giroux‐Metges:** Conceptualization; formal analysis; investigation; methodology; supervision; validation. **Erwan L'Her:** Conceptualization; investigation; methodology; project administration; supervision; validation. **François Guerrero:** Conceptualization; formal analysis; funding acquisition; investigation; methodology; project administration; supervision; validation.

## FUNDING INFORMATION

This research was funded by the Divers Alert Network under Grant DAN‐UBO‐2023‐GUE (to Prof François Guerrero) and by EG Dive and Research, Ind.

## CONFLICT OF INTEREST STATEMENT

The authors declared no conflict of interest.

## ETHICS STATEMENT

All procedures were conducted in accordance with the Declaration of Helsinki and were approved by the Committee for Protection of Persons Est I, Dijon, France (2022‐A01639‐34).

## CONSENT

Written informed consent was obtained from all individual participants included in the study.

## Supporting information


**Table S1:** Heart rate variability parameters (full data).
**Table S2:** Inflammatory biomarkers evolution during the follow‐up period (full data).

## Data Availability

Source data for this study are openly available at https://doi.org/10.6084/m9.figshare.32622399.
