# Supplementary material for: Heliox versus trimix in closed‐circuit rebreather diving: A comparative study of pulmonary and decompression stress in male divers
Source: Physiol Rep. 2026 Oct 5;14(19):e71129. doi: 10.14814/phy2.71129 (PMC13639384; doi:10.14814/phy2.71129)
Supplement: Supplementary file 1 [file PHY2-14-e71129-s001.docx]

## SUPPLEMENTAL MATERIAL

## Heliox and Trimix Physiological Impact on CCR Diving.

**Table S1.** Heart rate variability parameters (full data).

|  | Heliox | | | | Trimix | | | |
| --- | --- | --- | --- | --- | --- | --- | --- | --- |
|  | Pre | Post | D+1 | P-value | Pre | Post | D+1 | P-value |
| Time domain | | | | | | | | |
| Mean RR (ms) | 824 [765; 880] | 768 [676; 859] ^*^ | 863 [761; 914] | 0.03 | 853 [764; 940] | 778 [668; 813] ^†^ | 898 [793; 974] | 0.03 |
| SDNN (ms) | 34.3 [26.8; 44] | 41.4 [19.2; 51] | 32.2 [28.6; 38.5] | 0.4 | 47.7 [29.5; 82] | 49.4 [25.9; 91.9] | 56 [30.4; 72.3] | 0.97 |
| RMSSD (ms) | 25.5 [20.8; 31.2] | 27.9 [15.2; 31.2] | 21 [13.9; 31] | 0.7 | 36 [16.5; 61.5] | 35.6 [18.4; 68.8] | 35.1 [25.5; 63.9] | 0.7 |
| PNN50 (%) | 4.1 [1.23; 6.17] | 4.69 [0.98; 7.03] | 2.48 [0.3; 6.34] | 0.3 | 13.13 [1.01; 27.8] | 11.48 [1.54; 24.7] | 14.52 [4.26; 31.6] | 0.3 |
| Frequency domain | | | | | | | | |
| LF (ms^2^) | 600 [487; 1362] | 1171 [266; 2627] | 733 [469; 1077] | 0.97 | 1871 [642; 4819] | 1736 [490; 5777] | 1809 [438; 3889] | 0.7 |
| HF (ms^2^) | 214 [122; 233] | 214 [69; 319] | 145 [62; 254] | 0.6 | 427 [113; 1432] | 439 [111; 1243] | 453 [127; 1763] | 0.7 |
| LF/HF | 4.03 [2.46; 6.14] | 6.76 [3.41; 8.08] | 4.96 [2.7; 8.52] | 0.1 | 5.11 [2.62; 8.99] | 4.55 [3.34; 6.02] | 4.48 [1.1; 5.32] ^*^ | 0.046 |
| Non linear | | | | | | | | |
| SD1 (ms) | 18 [14.8; 20.3] | 19.7 [10.8; 21.9] | 14.9 [9.9; 21.9] | 0.7 | 25.5 [11.6; 43.5] | 25.2 [13.2; 48.7] | 24.8 [18; 45.2] | 0.7 |
| SD2 (ms) | 45.2 [34.2; 58.8] | 53.4 [25.5; 69.3] | 41.9 [38.1; 50.8] | 0.3 | 63 [40.2; 106.6] | 65 [34.8; 117.3] | 74.7 [38.8; 92] | 0.97 |
| DFA α1 | 1.32 [1.12; 1.53] | 1.48 [1.37; 1.6] | 1.37 [1.3; 1.57] | 0.3 | 1.41 [1.19; 1.53] | 1.39 [1.11; 1.52] | 1.31 [1.07; 1.44] | 0.7 |
| DFA α2 | 0.37 [0.34; 0.44] | 0.36 [0.3; 0.53] | 0.4 [0.32; 0.47] | 0.97 | 0.35 [0.3; 0.39] | 0.35 [0.27; 0.49] | 0.36 [0.29; 0.43] | 0.7 |
| ApEn | 1.24 [1.18; 1.3] | 1.1 [1.06; 1.28] ^††^ | 1.26 [1.2; 1.33] | 0.001 | 1.2 [1.1; 1.24] | 1.13 [0.91; 1.24] ^†^ | 1.24 [1.178; 1.31] | 0.03 |
| SampEn | 1.41 (1.32; 1.66] | 1.18 (1.01; 1.55] ^*^ | 1.59 (1.24; 1.64) | 0.02 | 1.36 (1.19; 1.49) | 1.14 (0.87; 1.44) ^†^ | 1.47 (1.39; 1.68) | 0.03 |
| Composite indexes | | | | | | | | |
| PNS | -1.07 [-1.3; -0.58] | -1.4 [-1.89; -0.81] | -0.97 [-1.37; -0.51] | 0.57 | -0.54 [-1.5; 0.37] | -0.93 [-1.8; 0.1] | -0.35 [-0.86; 0.26] | 0.09 |
| SNS | 0.86 [0.56; 1.2] | 1.44 [0.47; 2.52] | 0.63 [0.37; 1.76] | 0.28 | 0.32 [-0.46; 1.39] | 0.74 [0; 1.96] ^†^ | 0.25 [-0.46; 0.69] | 0.046 |

Results are expressed as median [IQR]. Intra-group comparisons were performed by Friedman test with Dunn’s post-hoc test. Post-hoc multiple comparisons test is expressed versus predive with * p < 0.05. Differences versus D+1 are expressed with † p < 0.05 and †† p < 0.01. SDNN: deviation of normal-to-normal R waves, RMSSD: root mean square of the successive difference, PNN50: proportion of number of successive intervals that differ more than 50ms, LF: low frequency, HF: high frequency, SD: standard deviation of Poincaré plot, DFA: detrended fluctuation analysis, ApEn: approximate entropy, SampEn: sample entropy, PNS: parasympathetic nervous system, SNS: sympathetic nervous system.

**Table S2.** Inflammatory biomarkers evolution during the follow-up period (full data).

|  | Heliox | | | | Trimix | | | |
| --- | --- | --- | --- | --- | --- | --- | --- | --- |
|  | Pre | Post | D+1 | P-value | Pre | Post | D+1 | P-value |
| MRP8/14 (µg.ml^-1^) | 1.68 [1.48; 2.43] | 4.56 [3.53; 6.74]^***^ | 2.37 [1.65; 3.39] | <0.0001 | 1.03 [0.6; 1.59] | 3.41 [1.63; 4.45]^**,††^ | 1.26 [0.71; 1.51] | 0.0003 |
| CCL2 (MCP1) (pg.ml^-1^) | 180 [160.4; 196.8] | 236.9 [167.5; 288.1]^††^ | 142.5 [90.9; 154.5]^**^ | 0.001 | 161.7 [129.8; 214.7] | 190.9 [116.4; 383.2] | 135.7 [90.9; 207.5] | 0.09 |
| IL1β (pg.ml^-1^) | 3.52 [1.48; 3.94] | 2.03 [0.57; 4.44] | 2.32 [0.95; 2.92] | 0.052 | 2.42 [1.4; 3.28] | 3.05 [2.09; 9] | 2.63 [0.37; 3.77] | 0.4 |
| IL6 (pg.ml^-1^) | 6.06 [4.1; 6.98] | 7.22 [4.04; 12.74] | 5.22 [2.69; 7.17] | 0.6 | 4.25 [2.1; 7.07] | 6.55 [3.58; 8.8]^*^ | 4.37 [1.64; 9.58] | 0.046 |
| IL8 (pg.ml^-1^) | 7.3 [6.53; 11.62] | 8.22 [6.21; 18.12] | 6.62 [5.1; 8.29] | 0.4 | 6.36 [4.39; 10.41] | 8.88 [6.34; 11.68] | 5.74 [3.44; 7.23] | 0.1 |
| CRP (mg.l^-1^) | 2.88 [1.54; 3.2] | 2.44 [1.57; 4.13]^†^ | 4.4 [3.3; 5.77] | 0.03 | 2.89 [1.38; 5.12] | 1.89 [1.41; 4.29] | 4.02 [1.71; 6.91] | 0.6 |
| MPO (ng.ml^-1^) | 138.6 [84.5; 194] | 143.3 [82.7; 176.8] | 166.7 [127.1; 224.8] | 0.3 | 118.8 [53.3; 139.2] | 117.4 [77.8; 157.5] | 123 [76.6; 155.4] | 0.08 |
| EGF (pg.ml^-1^) | 10 [2.19; 16.69] | 11.7 [5.07; 15.64] | 5.84 [4.84; 28.9] | 0.97 | 2.78 [1.41; 8,98] | 10.73 [7; 17.17] | 3.71 [1.34; 20.04] | 0.97 |
| NGAL (ng.ml^-1^) | 142.2 [98.03; 171.5] | 188.8 [131.8; 204.1] | 172.6 [112.2; 257.5] | 0.2 | 125.5 [83.1; 144] | 142.9 [123.7; 152.2] | 117.9 [90.3; 156.7] | 0.3 |
| iCAM1 (ng.ml^-1^) | 3.61 [2.42; 4.48] | 4.07 [3.52; 4.24] | 4.22 [3.27; 5.28] | 0.6 | 2.77 [1.55; 4.32] | 3.31 [2.45; 3.43] | 2.77 [2.32; 3.49] | 0.7 |
| VCAM1 (µg.ml^-1^) | 2.29 [2.16; 2.76] | 2.36 [2.03; 2.92] | 2.66 [2.19; 4.19] | 0.96 | 2.74 [1.73; 3.15] | 2.57 [2.05; 3.15] | 2.74 [2.25; 3.64] | 0.3 |
| Cystatin C (mg.l^-1^) | 0.48 [0.41; 0.5] | 0.39 [0.35; 0.43]^††^ | 0.48 [0.43; 0.67] | 0.005 | 0.43 [0.31; 0.52] | 0.42 [0.36; 0.57] | 0.47 [0.34; 0.53] | 0.3 |

Results are expressed as median [IQR]. Intra-group comparisons were performed by Friedman test with Dunn’s post-hoc test. Post-hoc multiple comparisons test is expressed versus predive with * p < 0.05 and ** p < 0.01 and *** p < 0.001. Differences versus D+1 are expressed with † p < 0.05 and †† p < 0.01.
